# Effect of medications and epidural steroid injections on fractures in postmenopausal women with osteoporosis

**DOI:** 10.1097/MD.0000000000016080

**Published:** 2019-06-28

**Authors:** Minsoo Kim, Yun-Ho Yang, Hee-Jeong Son, Jin Huh, Yuseon Cheong, Seong-Sik Kang, Byeongmun Hwang

**Affiliations:** Department of Anesthesiology and Pain Medicine, Kangwon National University Hospital, School of Medicine, Kangwon National University, Chuncheon, Gangwon-do, Republic of Korea.

**Keywords:** bone mineral density, epidural steroid injection, fracture, low back pain, osteoporosis, postmenopausal

## Abstract

Osteoporosis is a common problem, especially among postmenopausal women. Postmenopausal women with osteoporosis have major risk factors for osteoporotic fractures. The abuse of epidural steroid injections (ESIs) or the misunderstanding of their proper use could cause osteoporotic fractures. Therefore, we aimed to investigate whether ESIs are associated with osteoporotic fractures in postmenopausal women with low back pain and osteoporosis. Furthermore, we aimed to provide evidence on whether ESIs could be used in postmenopausal women with osteoporosis who are at high risk for osteoporotic fractures.

We reviewed the medical records of postmenopausal women with osteoporosis but no fractures. A total of 172 postmenopausal women were divided into 2 groups. Group 1 comprised patients receiving medications and Group 2 comprised patients receiving ESIs. All participants received medications for treating osteoporosis. Each patient's age, bone mineral density, body mass index, medical history, and status with respect to smoking, drinking, physical activity, and exercise were obtained using a questionnaire and medical records.

The mean total number of ESIs was 6.2, and the mean cumulative administered dose of glucocorticoids (dexamethasone) was 31 mg. The incidences of fractures in the medication and ESI groups were 22% and 24%, respectively, in the thoracolumbar spine, and 2% and 5%, respectively, in the hip joint.

There was no significant difference in the incidences of osteoporotic fractures at the thoraco-lumbar spine and hip joint in postmenopausal women with osteoporosis between those who received ESIs (a mean of 6.2 ESIs, a cumulative dexamethasone dose of 31 mg) and those who did not, with both groups taking anti-osteoporotic medications for low back pain. Our data suggest that ESI treatment using a mean of 6.2 ESIs to deliver a maximum cumulative dexamethasone dose of 31 mg could be safely used in postmenopausal women with osteoporosis, without any significant impact on the their risk for osteoporotic fractures.

## 1 Introduction

Epidural steroid injections (ESIs) are commonly used in the management of low back pain and radiculopathy.^[1–3]^ There has been an increase in the use of ESIs, which can be attributed to the avoidance of either more invasive surgery or addictive medications. However, the increased use of ESIs could also be because of a misunderstanding of the indications for its proper use, which may lead to abuse of this procedure. Recently, there have been reports on the complications of ESIs.^[4,5]^ The opinion that the use of ESIs should be limited to prevent complications, such as avascular necrosis of joints, hyperglycemia, osteoporosis, and vertebral compression fractures, is gaining strength among physicians.^[6]^

Fractures associated with osteoporosis lead to impairment in mobility and an increase in mortality.^[7,8]^ The major risk factors for osteoporotic fractures are female sex, old age, low bone mineral density (BMD), and history of previous fractures.^[9]^ Osteoporosis is a common problem, especially among postmenopausal women.^[9,10]^ Most of the major risk factors mentioned above are present in postmenopausal women with osteoporosis. Furthermore, patients with low back pain may have low BMD because they engage in less physical activity,^[11,12]^ or because of the frequent use of ESIs.^[13]^ Therefore, practitioners must pay attention to fracture risk when they administer ESIs to postmenopausal women with low back pain. Based on these factors, it is important to evaluate the relationship between ESIs and osteoporotic fractures in postmenopausal women with low back pain.

ESIs can have a positive effect on the prevention of osteoporotic fractures because pain relief can lead to an increase in physical activity and make it easier to exercise. However, the glucocorticoids used in ESIs can decrease the BMD and increase fracture risk. However, the relationship between osteoporotic fractures and ESI has not been thoroughly investigated, and the available results are inconsistent. The risk of osteoporotic fractures in postmenopausal women is high; therefore, the effect of ESI on postmenopausal women with osteoporosis needs to be thoroughly investigated.

In the present study, we aimed to investigate whether ESI is associated with osteoporotic fractures in postmenopausal women with low back pain and osteoporosis. Further, we aimed to evaluate whether ESI could be used in postmenopausal women with osteoporosis who are at high risk for osteoporotic fractures.

## 2 Methods

### 2.1 Study population

This study was a retrospective analysis of postmenopausal women who had received either ESIs or medication for low back pain in the Kangwon National University Hospital (Gangwon-do, South Korea) between January 2006 and December 2012. The study was conducted according to the Declaration of Helsinki^[14]^ and was approved by the local institutional review board (KNUH-2018 -05-010).

The study participants included postmenopausal women with osteoporosis who had received either ESIs or medication for low back pain. The inclusive criteria were no history of osteoporotic fractures, having undergone radiography and BMD assessments within a year before receiving ESI or medication, and having radiographic films and medical records for up to 5 years after receiving ESI or medication for low back pain. The exclusion criteria were a history of co-morbidities known to affect bone metabolism, such as cancer, pituitary diseases, thyroid disease, rheumatic disease, renal failure, or adrenal disease; previous osteoporotic fracture; fractures owing to known accidental traumas; and lumbar spine or femoral surgery.

We selected a total of 172 patients aged ≥55 years and older as participants who satisfied the inclusion criteria after age (±1 year) and body mass index (BMI) matching. The study participants were divided into 2 groups, with 86 participants each. Group 1 consisted of patients who received medications and Group 2 consisted of patients who received ESIs. The treatments of osteoporosis in all the participants included bisphosphonates, calcium, and hormone replacement therapy. Based on a questionnaire, details about age at menopause, medical history, and status concerning smoking, drinking, physical activity, and exercise were recorded for each study participant. Physical activity was categorized into 3 groups: low (absence of moderate or vigorous activity weekly); moderate (moderate activity at least once per week); and vigorous (vigorous activity at least once per week).^[13]^ The occupations of the participants were not considered as most of them were housewives and did not hold other jobs.

The ESIs, consisting of a mixture of 8 mL lidocaine hydrochloride (0.5%, preservative free) and dexamethasone, were administered at the lumbar spine levels. The procedures were fully explained and written informed consent was obtained before the procedure. All the procedures were performed by expert staff, under fluoroscopic guidance, in a sterile ambulatory surgical setting, and with appropriate monitoring. All participants initially received 1–3 ESIs at 2-week intervals. Additional lumbar ESIs were provided based on the participant's response to previous injections.

The results of BMD measurements were recorded, as well as details of any fragility fractures, the anatomical site involved, and the treatment administered. The BMD of the lumbar spine (L1–L4), femoral neck, and total femur was measured by dual energy x-ray absorptiometry using Lunar Prodigy (DXA; GE Healthcare, WI) and expressed as absolute values (gram per square centimeters). The BMD values were also expressed as T-scores. T-scores for the bone were calculated by taking the difference between the measured BMD and the mean BMD of healthy young adult Korean women (age, 20–40 years) matched for sex and ethnicity, divided by the standard deviation (SD) of the young adult population. Osteoporosis was defined as a T score <−2.5 and osteopenia as a T score >−2.5 but <−1.0 according to the World Health Organization criteria.^[15]^

The anterior–posterior and lateral radiographs of the thoracic and lumbar spine showing presence of vertebral fractures were interpreted by radiographic morphometry using Genant semiquantitative method.^[16]^

### 2.2 Sample size

The sample size was determined on the basis of previous assessments and studies.^[17,18]^ The primary outcome was the incidence of fractures in patients with osteoporosis. Considering a 0.05 2-sided significance level, a power of 90%, an allocation ratio of 1:1, and an effect size of 0.5, 86 patients in each group were estimated.

### 2.3 Statistical analysis

The data were presented as the mean ± standard deviation (SD). The mean comparisons of the demographic and clinical data between the 2 groups were made using Student *t* test. The *χ*^2^ test and Fisher exact test were used to compare the differences between group proportions.

In all the comparisons, a *P* value of <0.05 was considered statistically significant. Statistical analyses were performed using SPSS 23.0 (IBM Corporation, Somers, NY).

## 3 Results

The participants’ characteristics are presented in Table 1. No significant differences in age, weight, and height were found between the 2 groups. The BMD of the lumbar spine, femoral neck, and total femur were similar in both groups. The mean total number of ESIs was 6.2, and the mean cumulative administered dose of corticosteroids (dexamethasone) was 31 mg.

**Table 1:**
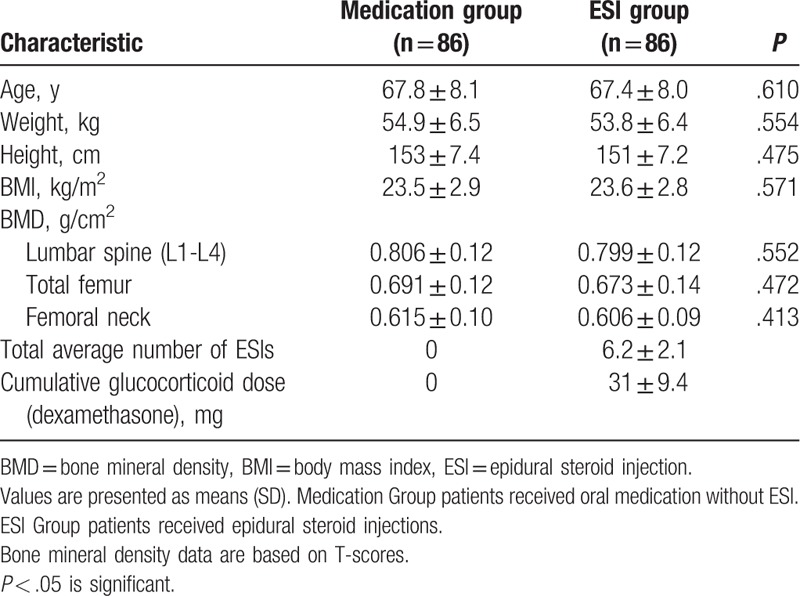
Baseline demographic and clinical characteristics.

The prevalence of fractures in patients with osteoporosis is presented in Table 2. The incidence of fractures in the medication group and the ESI group was 22% and 24% at the thoracolumbar spine, and 2% and 5% at the hip joint, respectively.

**Table 2:**
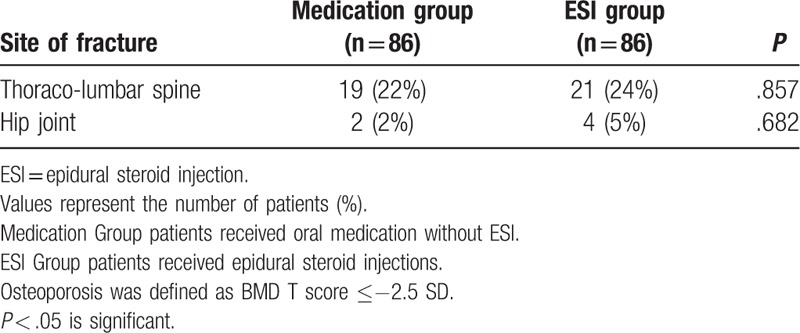
Prevalence of fracture in postmenopausal women with osteoporosis.

The number of patients who developed osteoporotic fractures of the thoracolumbar spine at the 1- to 5-year follow-up is presented in Table 3. The number of patients who developed osteoporotic fractures at the hip joint at the 1- to 5-year follow-up is presented in Table 4. There were no significant differences in the prevalence of osteoporotic fracture between the 2 groups for any follow-up period.

**Table 3:**
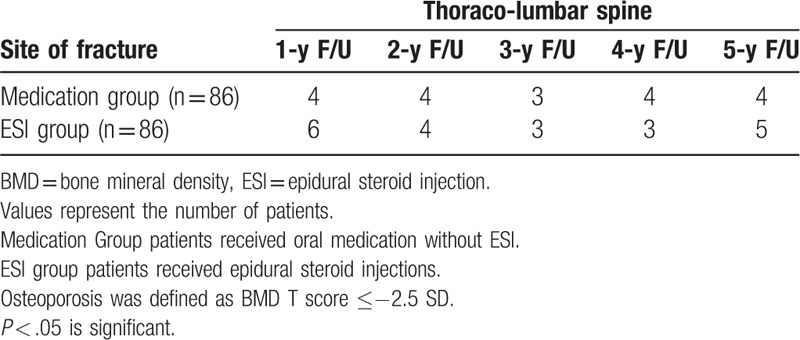
Prevalence of fracture at the thoracolumbar spine in 5 years follow-up in patients with osteoporosis.

**Table 4:**
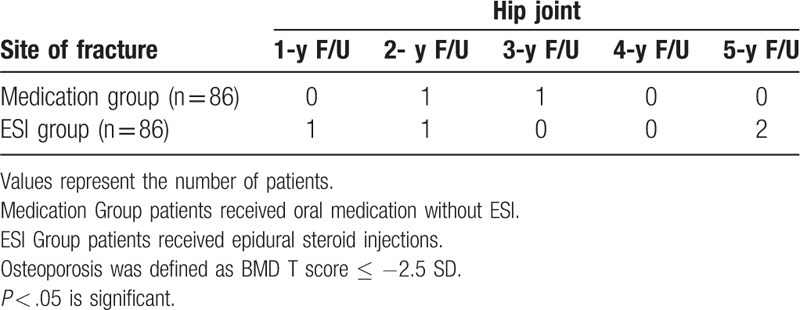
Prevalence of fracture at the hip joint in 5 years follow-up in patients with osteoporosis.

The lifestyle characteristics of the participants are listed in Table 5. There were no statistically significant differences between the 2 groups based on smoking, alcohol drinking, exercise, or physical activity.

**Table 5:**
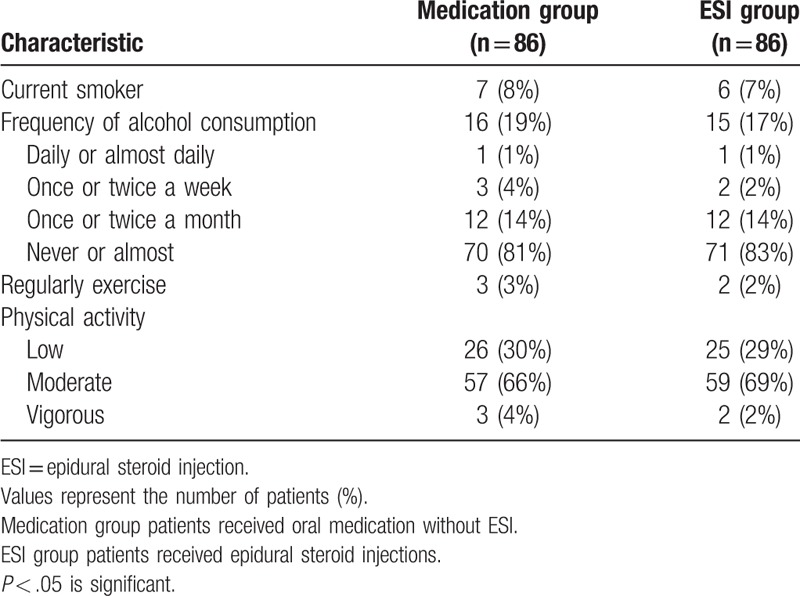
Lifestyle characteristics of the patients.

## 4 Discussion

The present study analyzed the incidence of osteoporotic fractures in a 5-year period in postmenopausal women with osteoporosis and low back pain who had received either ESIs or medication. The results show that under similar conditions of age, BMI, BMD, and lifestyle, there was no significant difference between the 2 groups in the incidence of osteoporotic fractures at the thoracolumbar spine and hip joint.

Glucocorticoids have multiple adverse effects.^[6,10]^ Osteoporosis is a major complication of glucocorticoid treatment.^[6,10]^ Greater cumulative glucocorticoid administration (triamcinolone 400 mg) might be associated with decreased BMD,^[13]^ which is a major determinant of the risk of osteoporotic fractures.^[19,20]^ Therefore, the risk of osteoporotic fractures appears to increase with glucocorticoid therapy.^[21,22]^ However, studies on the effects of ESIs on the BMD and fractures have produced inconsistent results.^[5,22,23]^ Manchikanti et al^[24]^ reported that low doses of ESIs are safe and do not affect BMD. Some other studies have also found no significant relationship between ESI, BMD, and vertebral fractures at low cumulative doses of glucocorticoids.^[23,25,26]^ In contrast, some recent reports have shown that ESI negatively influences BMD.^[4,13,18]^ Kim et al^[13]^ reported that frequent ESIs (mean number of 14, mean total dose of triamcinolone 400 mg) might reduce BMD in postmenopausal women with low back pain. Some studies reported that administration of exogenous glucocorticoids reduces BMD and increases the risk of fractures.^[22,27,28]^ Glucocorticoid-induced bone loss is reported to be associated with the dose and duration.^[19–22]^ However, the majority of studies, excluding those on higher cumulative doses of glucocorticoids, did not find a relationship between ESIs and osteoporosis and osteoporotic fractures.^[23–26]^

In postmenopausal women who do not take antiosteoporotic medications, ESIs were found to be correlated with significant BMD changes in the femoral neck.^[18]^ Nah et al^[17]^ reported that the BMD after ESIs in patients taking antiosteoporotic medications recovered within 2 years. Other studies have suggested that old age and a low baseline BMD, rather than ESIs, are associated with increased risk of osteoporotic fractures in postmenopausal women with low back pain treated with ESI.^[23,24,26]^ In the present study, all the patients were receiving antiosteoporotic medications for the treatment of osteoporosis. The mean of ESI administration was 6.2 times and the cumulative dose of glucocorticoid (dexamethasone) was 31 mg. Although there was a decrease in the BMD in the ESI group, there was no difference in the incidence of osteoporotic fractures between the medication group and the ESI group. This is probably because of the combination of antiosteoporotic medication use, the relatively low number of ESIs administered, and the low doses of corticosteroids used.

In a study of postmenopausal women who had received ESIs for low back pain, Nah et al^[17]^ reported that there were no significant differences in the incidence of spine and hip fractures between patients who received ESIs and those who did not. Similarly, we observed that there was no significant difference in the incidence of fractures of the thoracolumbar spine and the hip joint between the medication group and the ESI group. However, our study has certain advantages over the study by Nah et al.^[17]^ First, our study excluded patients with preexisting fractures because these are a common cause of newly occurring fractures. Second, participants of the present study included only postmenopausal women with osteoporosis and hence have a higher risk of osteoporotic fractures. Third, our study used data collected over a relatively longer period (5 years) than that in other studies. Therefore, our study is more beneficial in providing evidence that ESI could be administered to postmenopausal women with osteoporosis.

The prevalence of vertebral fractures in the elderly population is estimated to be at least 20%.^[29]^ Kim et al^[30]^ reported that the incidence of vertebral fractures was 21% in healthy postmenopausal women between the ages of 60 and 79 years (mean, 65.1 years) in South Korea. In another study,^[17]^ the prevalence of newly occurring vertebral fractures in postmenopausal women treated with ESIs for low back pain was 22% during the 2 years’ follow-up. In the present study, the incidences of thoracolumbar spine fractures in the postmenopausal women with osteoporosis during the 5-year follow-up were 45% and 56% in the medication group and ESI group, respectively. Yi et al^[23]^ reported that the incidence of hip joint fracture was 8% in postmenopausal women who received ESIs. In the present study, the incidences of hip joint fractures during the 5-year follow-up were 2% and 5% in the medication and ESI groups, respectively. The high incidence of osteoporotic fractures in the present study may be because of osteoporosis and lower physical activity in addition to old age and female sex. In this study, the percentage of patients who regularly exercised was 3% and 2% in the medication group and ESI groups, respectively. Moreover, the percentage of patients who did vigorous physical activity was 4% and 2% in the medication group and ESI group, respectively. In the present study, the lower physical activity of the patients may be attributed to low back pain, old age, and the sociocultural environment.

In the present study, the incidences of thoracolumbar spine fractures were 45% and 56% in the medication group and ESI group, respectively. The incidences of hip joint fractures were 2% and 5% in the medication group and ESI group, respectively. Furthermore, there were fewer fractures, although not significantly, in the medication group, probably because of the lower BMDs, although not significantly, in the ESI group.

The lowering of BMD and the onset of fracture risk appears to be very rapid, with the maximum risk of fracture occurring within 6 months of starting external glucocorticoid therapy.^[17,18,21]^ However, a reduction of fracture risk has been seen after 1 year of discontinuing therapy with glucocorticoids. At 2-year follow-up, reduction in the BMD is reversible following the interruption of glucocorticoid treatment.^[17–21]^ In the present study, the incidence of osteoporotic fractures was not significantly affected by the post-ESI year period. Therefore, it is possible that the decrease in BMD following ESIs was only slightly because of the use of antiosteoporosis medication. It is also possible that there was an increase in the physical activity undertaken by the patient owing to decreased pain following the administration of ESIs, with most patients reporting increased external activity and walking distance owing to improvements in walking and reduced pain after receiving ESIs. However, we could not measure the increase in physical activity objectively.

Osteoporotic fractures are associated with deterioration in the quality-of-life and increased mortality.^[29]^ Accordingly, the impact of ESIs on BMD and skeletal health must be carefully considered, and patients should be made aware of the potential increase in the osteoporotic fracture risk with each additional injection. Many patients receiving ESIs are old and seek frequent injections to maintain an active lifestyle and/or avoid undergoing surgery. Furthermore, postmenopausal women with osteoporosis have major risk factors for osteoporotic fractures, such as female sex, old age, and a low BMD. These patients are more likely to have compromised skeletal quality, putting them at a greater risk for osteoporotic fractures and compromised BMD from exogenous glucocorticoid treatment.^[22]^ Therefore, practitioners must pay attention to osteoporotic fracture risk when treating low back pain using ESIs in postmenopausal women with osteoporosis. Moreover, monitoring of BMD and therapeutic intervention at adequate intervals should be considered for reducing the risk of osteoporotic fractures when ESIs are administered, particularly in patients with additional risk factors for osteoporotic fractures. In light of the results of this study, the use of glucocorticoids at a low dose through few ESIs can be observed not to have a significant effect on the incidence of osteoporotic fractures in postmenopausal women with osteoporosis were receiving antiosteoporotic medications. However, glucocorticoid-induced bone loss is dose and duration related.^[19–22]^ Therefore, frequent ESIs and use of higher doses of glucocorticoids may increase the incidence of osteoporotic fractures. However, if recent guidelines for glucocorticoid use are followed, ESIs are unlikely to increase the incidence of osteoporotic fractures.^[31–33]^

This study had some limitations. First, this study had a retrospective design. However, to reduce any possible selection bias, our inclusion criteria were restrictive. Furthermore, the authors matched sex, age, and history of osteoporotic fractures in participants to increase the accuracy of the analysis. Second, all the patients took anti-osteoporotic medication, which could obscure the adverse effects of ESIs. However, antiosteoporotic medications are routinely prescribed to patients with osteoporosis to improve BMD or prevent osteoporosis-related fractures. Third, our study is a single-institution observational study with considerable heterogeneity and limited generalizability. Further studies can consider prospective randomized controlled trials to investigate the effect of ESIs on osteoporotic fractures in postmenopausal women.

## 5 Conclusion

There was no significant difference in the incidences of osteoporotic fractures at the thoracolumbar spine and hip joint in postmenopausal women with osteoporosis between those who received ESIs (a mean of 6.2 ESIs, a cumulative dexamethasone dose of 31 mg) and those who did not, with both groups taking anti-osteoporotic medications for low back pain. Our data suggest that ESI treatment using a mean of 6.2 ESIs to deliver a maximum cumulative dexamethasone dose of 31 mg could be safely used in postmenopausal women with osteoporosis, without any significant impact on the their risk for osteoporotic fractures.

## Author contributions

**Conceptualization:** Minsoo Kim, Byeongmun Hwang.

**Data curation:** Yun-Ho Yang, Yuseon Cheong, Seong-Sik Kang.

**Formal analysis:** Hee-Jeong Son, Jin Huh, Seong-Sik Kang.

**Investigation:** Yun-Ho Yang.

**Methodology:** Minsoo Kim, Byeongmun Hwang.

**Resources:** Yuseon Cheong.

**Supervision:** Byeongmun Hwang.

**Writing – original draft:** Byeongmun Hwang.

**Writing – review & editing:** Byeongmun Hwang.

Byeongmun Hwang orcid: 0000-0002-2795-0538.

## References

[1] Lee JH Shin KH Park SJ Comparison of clinical efficacy between transforaminal and interlaminar epidural injections in lumbosacral disc herniation: a systematic review and meta-analysis. Pain Physician 2018;21:433–48. 30282389

[2] Kim MJ Choi YS Suh HJ Unintentional lumbar facet joint injection guided by fluoroscopy during interlaminar epidural steroid injection: a retrospective analysis. Korean J Pain 2018;31:87–92. 29686806 10.3344/kjp.2018.31.2.87 PMC5904352

[3] Seo DK Lee S Lee G Retrodiscal epidural balloon adhesiolysis through Kambin's triangle in chronic lumbar spinal stenosis: A retrospective analysis and technical considerations. Medicine (Baltimore) 2018;97:e12791. 30313103 10.1097/MD.0000000000012791 PMC6203470

[4] Al-Shoha A Rao DS Schilling J Effect of epidural steroid injection on bone mineral density and markers of bone turnover in postmenopausal women. Spine (Phila Pa 1976) 2012;37:E1567–71. 23196966 10.1097/BRS.0b013e318270280e

[5] Mandel S Schilling J Peterson E A retrospective analysis of vertebral body fractures following epidural steroid injections. J Bone Joint Surg Am 2013;95:961–4. 23780532 10.2106/JBJS.L.00844

[6] Bellini M Barbieri M Systemic effects of epidural steroid injections. Anaesthesiol Intensive Ther 2013;45:93–8. 23877903 10.5603/AIT.2013.0021

[7] Cannada LK Hill BW Osteoporotic hip and spine fractures: a current review. Geriatr Orthop Surg Rehabil 2014;5:207–12. 26246944 10.1177/2151458514548579 PMC4252159

[8] Nazrun AS Tzar MN Mokhtar SA A systematic review of the outcomes of osteoporotic fracture patients after hospital discharge: morbidity, subsequent fractures, and mortality. Ther Clin Risk Manag 2014;10:937–48. 25429224 10.2147/TCRM.S72456 PMC4242696

[9] Orimo H Nakamura T Hosoi Iki M Japanese 2011 guidelines for prevention and treatment of osteoporosis—executive summary. Arch Osteoporos 2012;7:3–20. 23203733 10.1007/s11657-012-0109-9 PMC3517709

[10] Frenkel B White W Tuckermann J Glucocorticoid-induced osteoporosis. Adv Exp Med Biol 2015;872:179–215. 26215995 10.1007/978-1-4939-2895-8_8 PMC5905346

[11] Kemmler W Bebenek M Kohl M Exercise and fractures in postmenopausal women. Final results of the controlled Erlangen Fitness and Osteoporosis Prevention Study (EFOPS). Osteoporos Int 2015;26:2491–9. 25963237 10.1007/s00198-015-3165-3

[12] Muir JM Ye C Bhandari M The effect of regular physical activity on bone mineral density in post-menopausal women aged 75 and over: a retrospective analysis from the Canadian multicentre osteoporosis study. BMC Musculoskelet Disord 2013;14:253. 23971674 10.1186/1471-2474-14-253 PMC3765292

[13] Kim S Hwang B Relationship between bone mineral density and the frequent administration of epidural steroid injections in postmenopausal women with low back pain. Pain Res Manag 2014;19:30–4. 24404559 10.1155/2014/870145 PMC3938340

[14] Krleža-Jerić K Lemmens T 7th Revision of the Declaration of Helsinki: good news for the transparency of clinical trials. Croat Med J 2009;50:105–10. 19399942 10.3325/cmj.2009.50.105 PMC2681053

[15] Assessment of fracture risk and its application to screening for postmenopausal osteoporosis. Report of a WHO Study Group. World Health Organ Tech Rep Ser 1994;843:1–29. 7941614

[16] Ferrar L Jiang G Adams J Identification of vertebral fractures: an update. Osteoporos Int 2005;16:717–28. 15868071 10.1007/s00198-005-1880-x

[17] Nah SY Lee JH Lee JH Effects of epidural steroid injections on bone mineral density and bone turnover markers in patients taking anti-osteoporotic medications. Pain Physician 2018;21:E435–47. 30045610

[18] Kim YU Karm MH Cheong Y Effect of epidural steroid injection on bone mineral density in postmenopausal women according to antiosteoporotic medication use. Pain Physician 2016;19:389–96. 27454269

[19] Compston J Glucocorticoid-induced osteoporosis: an update. Endocrine 2018;61:7–16. 29691807 10.1007/s12020-018-1588-2 PMC5997116

[20] Maroutti N Corrado A Cantatore FP Glucocorticoids induced risk of fractures. Panminerva med 2010;52:339–43. 21183894

[21] van Staa TP Leufkens HG Abenhaim L Oral corticosteroids and fracture risk: relationship to daily and cumulative doses. Rheumatology 2000;39:1383–9. 11136882 10.1093/rheumatology/39.12.1383

[22] Kerezoudis P Rinaldo L Alvi MA The effect of epidural steroid injections on bone mineral density and vertebral fracture risk: a systematic review and critical appraisal of current literature. Pain Med 2018;19:569–79. 29304236 10.1093/pm/pnx324

[23] Yi Y Hwang B Son H Low bone mineral density, but not epidural steroid injection, is associated with fracture in postmenopausal women with low back pain. Pain Physician 2012;15:441–9. 23159959

[24] Manchikanti L Pampati V Beyer C The effect of neuraxial steroids on weight and bone mass density: a prospective evaluation. Pain Physician 2000;3:357–66. 16906177

[25] Kang SS Hwang BM Son H Changes in bone mineral density in postmenopausal women treated with epidural steroid injections for lower back pain. Pain Physician 2012;15:229–36. 22622907

[26] Dubois EF Wagemans MF Verdouw BC Lack of relationships between cumulative methylprednisolone dose and bone mineral density in healthy men and postmenopausal women with chronic low back pain. Clin Rheumatol 2003;22:12–7. 12605311 10.1007/s10067-002-0648-3

[27] van Staa TP The pathogenesis, epidemiology and management of glucocorticoid- induced osteoporosis. Calcif Tissue Int 2006;79:129–37. 16969593 10.1007/s00223-006-0019-1

[28] Osella G Ventura M Ardito A Cortisol secretion, bone health, and bone loss: a cross-sectional and prospective study in normal nonosteoporotic women in the early postmenopausal period. Eur J Endocrinol 2012;166:855–60. 22312036 10.1530/EJE-11-0957

[29] Puisto V Rissanen H Heliö vaara M Vertebral fracture and cause-specific mortality: A prospective population study of 3, 210 men and 3, 730 women with 30 years of follow-up. Eur Spine J 2011;20:2181–6. 21611851 10.1007/s00586-011-1852-0 PMC3229726

[30] Shin CS Kim MJ Shim SM The prevalence and risk factors of vertebral fractures in Korea. J Bone Miner Metab 2012;30:183–92. 21773702 10.1007/s00774-011-0300-x

[31] Compston J Cooper A Cooper C National Osteoporosis Guideline Group (NOGG). UK clinical guideline for the prevention and treatment of osteoporosis. Arch Osteoporos 2017;12:43. 28425085 10.1007/s11657-017-0324-5 PMC5397452

[32] Manchikanti L Datta S Gupta S A critical review of the American Pain Society clinical practice guidelines for interventional techniques: part 2. Therapeutic interventions. Pain Physician 2010;13:E215–64. 20648212

[33] Buckley L Guyatt G Fink HA 2017 American College of Rheumatology Guideline for the prevention and treatment of glucocorticoid-induced osteoporosis. Arthritis Care Res (Hoboken) 2017;69:1521–37. 10.1002/art.40137 28585373

